# B cell immunofocusing and repriming in two HIV-1 Env immunization regimens

**DOI:** 10.21203/rs.3.rs-3895128/v1

**Published:** 2024-04-08

**Authors:** Jenna M. DeLuca, Maria Blasi, Shalini Jha, Xiaoying Shen, Justin Pollara, Melissa Kerkau, Mansi Purwar, Diane G. Carnathan, Donatella Negri, Andrea Cara, Kurt Wollenberg, Kevin O. Saunders, Shan Lu, Guido Silvestri, David B. Weiner, Mary E. Klotman, Guido Ferrari, M. Anthony Moody, Mattia Bonsignori

**Affiliations:** 1 Translational Immunobiology Unit, Laboratory of Infectious Diseases, National Institute of Allergy and Infectious Diseases, National Institutes of Health, Bethesda, MD, USA.; 2 Duke Human Vaccine Institute, Duke University, Durham, NC, USA.; 3 Department of Medicine, Duke University, Durham, NC, USA.; 4 Department of Surgery, Duke University, Durham, NC, USA.; 5 Wistar Institute, Philadelphia, PA, USA.; 6 Emory National Primate Research Center and Emory Vaccine Center, Emory University School of Medicine, Atlanta, GA, USA.; 7 Department of Infectious Diseases, Istituto Superiore di Sanità, Rome, Italy.; 8 National Center for Global Health, Istituto Superiore di Sanità, Rome, Italy.; 9 Bioinformatics & Computational Biosciences Branch, National Institute of Allergy and Infectious Diseases, National Institutes of Health, Bethesda, MD, USA.; 10 University of Massachusetts Medical School, Worcester, MA, USA.; 11 Department of Pediatrics, Duke University, Durham, NC, USA.

## Abstract

Diverse and rapidly mutating viruses pose challenges to immunogen and vaccine design. In this study, we evaluated the ability of memory B-cells obtained from two independent NHP trials to cross-react with individual HIV-1 vaccine components of two different multivalent immunization strategies. We demonstrated that while an HIV-1 Env multiclade, multivalent immunization regimen resulted in a dominant memory B-cell response that converged toward shared epitopes, in a sequential immunization with clonally-related non-stabilized gp140 HIV-1 Envs followed by SOSIP-stabilized gp140 trimers, the change in immunogen format resulted in repriming of the B-cell response.

Successful vaccines against highly mutating viruses, such as influenza virus and SARS-CoV-2, rely on sequential immunizations with mono- or multivalent formulations to counter viral diversity and broaden the spectrum of primed B-cells at the time of exposure.^1^ For HIV-1, multivalent, multiclade vaccination schemes - a method historically used to counter viral diversity - and sequential immunization strategies with clonally related HIV-1 envelope (Env) glycoproteins have been pursued.^2^ Despite intensive studies, the relative frequency of immunogen mono- *versus* cross-reactive B-cells in repeated immunizations is not fully characterized. Here, we analyzed the cross-reactivity of B-cells isolated from non-human primates (NHPs) immunized with either a multiclade vaccine formulation or sequential immunizations with clonally related non-stabilized gp140 Envs followed by SOSIP stabilized gp140 trimers.

In the first study, four macaques (RNs14, RVv15, RPt15 and RSf15) were primed with DNA and boosted with a tetravalent HIV-1 gp120 Env cocktail comprising clades A, B, C and CRF AE strains (Figure 1a). Upon heterologous SHIV challenge, all but one macaque (RVv15) was infected (Figure 1b). No neutralization against tier 2 viruses was observed. Memory B-cells that bound to the tetravalent cocktail of Env proteins were sorted from PBMCs collected two weeks post-final boost and cultured as described.^3^ For each B-cell culture supernatant, binding to individual immunogens was assessed. In total, we analyzed 734 Env-reactive B-cells (RNs14: n=34, RVv15: n=208, RPt15: n= 237, RSf15: n=255). Only 143/734 (19%) reacted with a single Env immunogen (Figure 1c). Mono-specificity was predominantly directed to B.JR-FL (n=80, 56%), followed by C.93MW965.26 (n=31, 22%), AE consensus (n=25, 17%), and A.92UG037.1 (n=7, 5%), with similar distributions across the four macaques (Figure 1d). Of the 591 B-cells that bound to multiple Envs, the majority (n=327/591; 55%) reacted with all four immunogens (RNs14: n=17/28, 61%; RVv15: n=79/154, 51%; RPt15: n=106/209, 51%; RSf15: n=125/200, 63%) (Figure 1e). Thus, the B-cells expanded by this tetravalent multiclade formulation predominantly converged toward shared epitopes.

In the second study, macaques were immunized sequentially with gp140 Envs isolated from individual CH505 (transmitted founder virus [TF] and variants isolated from weeks 53, 76, 100 and 136 post-infection).^4^ Immunogens were delivered via an integrase-defective lentiviral vector (IDLV) either alone or with the respective protein (Figure 2a).^4^ The first four immunogens were administered as non-stabilized gp140 Envs, whereas CH505.wk136 was administered as stabilized gp140 SOSIP Env trimer intended to focus antibody responses toward broadly neutralizing epitopes.^4^ Upon autologous SHIV challenge, no neutralization against tier 2 viruses was observed. All but one macaque (Rh6601, IDLV+protein group) was infected, with two macaques (Rh6600, IDLV+protein group and Rh6575, IDLV only group) resisting infection up to the tenth challenge.^4^ In these three macaques, we measured the frequency of Env immunogen-specific memory B-cell responses after the last non-stabilized gp140 Env and SOSIP administrations (weeks 75 and 117, respectively). From week 75, we analyzed a total of 410 Ig+ culture supernatants from immunogen-reactive B-cells (Rh6601: n=79, Rh6600: n=54, Rh6575: n=277). Most B-cells (n=331/410, 81%) cross-reacted with all gp140 Env immunogens (Rh6601: n=64/79, 81%; Rh6600: n=39/54, 72%; Rh6575: n=228/277, 82%) and <6% reacted with a single gp140 Env (Rh6601: n=4, 5.1%; Rh6600: n=3, 5.6%; Rh6575: n=15, 5.4%) (**Figure S1** and Figure 2b). Among the gp140 Env-reactive B-cells, 27% also reacted with trimeric CH505.wk136 SOSIP (Rh6601: n=22/79, 28%; Rh6600: n=11/54, 20%; Rh6575: n=76/277, 27%), which was not yet administered at week 75 (Figure 2b). The vast majority (95%) of the trimer-binding B-cells reacted with all four gp140 Env immunogens (Figure 2b). Hence, sequential immunization with non-stabilized gp140 Envs expanded B-cells that cross-reacted with and were available for engagement to the trimer. In line with the multivalent, multiclade study described above, these results demonstrate that immunization with clonally related gp140 Envs elicited a dominant B-cell response that converged toward epitopes shared across the immunogens.

After CH505.wk136 gp140 SOSIP Env trimer boosting (week 117), trimer-reactive cells increased from 27% to 86% (All NHPs: 219/256; Rh6601: 159/163, 98%; Rh6600: 31/62, 50%; Rh6575: 29/31, 94%) (Figure 2c). We note that the CH505.wk136 SOSIP trimer ELISA for Rh6600 had high background, which likely resulted in an underestimate of trimer-reactive cultures for this macaque. Differently from what was observed after immunization with non-stabilized gp140 Envs, the majority of CH505.wk136 SOSIP trimer-reactive B-cells (179/219, 82%) did not cross-react with the preceding gp140 Env immunogens (Rh6601: 141/159, 89%; Rh6600: 17/31, 55%; Rh6575: 21/29, 72%) (Figure 2c). Thus, the change in immunogen design from non-stabilized gp140 to SOSIP-stabilized trimeric Env induced substantial B-cell repriming.

In summary, this study exemplifies how the choice of immunogen designs can affect the engagement of different B-cell pools in the context of sequential immunizations: In particular, how they can alter the balance between B-cell convergence toward shared epitopes (i.e., immunofocusing) and B-cell repriming. Specifically, despite the genetic distance among immunogens in the multiclade immunization scheme (**Figure S2**), the observed cross-clade breadth was predominantly mediated by B-cells immunofocused on shared epitopes, rather than by an additive engagement of multiple clade-specific B-cells. Immunofocusing was unsurprisingly more pronounced in sequential immunizations with clonally related non-stabilized gp140 Envs. Non-stabilized gp140 Env immunization expanded a pool of B-cells for which the cognate epitopes were accessible on the surface of SOSIP-stabilized gp140 trimers. However, despite these cross-reactive B-cells being present, the change in immunogen from non-stabilized gp140 to SOSIP-stabilized gp140 Env largely bypassed their re-engagement and induced substantial B-cell repriming of a different B-cell pool that did not cross-react with the previously administered gp140 Envs.

The minimal engagement of these pre-existing cross-reactive B-cells implies that the epitope immunodominance hierarchy of the SOSIP trimer differed from that of the non-stabilized gp140 Envs. The stabilized SOSIP trimer design used in this study was templated on the BG505 SOSIP.664 trimer backbone, which displays a dominant neo-epitope cluster at the base region of the trimer.^5,6^ This dominant neo-epitope may have contributed to the observed B-cell repriming by SOSIP immunization in our study, supporting the development of strategies to silence undesired responses against the SOSIP base.^7,8^ Critically, and regardless of the specific epitopes involved, we demonstrate that B-cell priming with non-stabilized Env was insufficient to modify the effect of a different epitope immunodominance hierarchy in a SOSIP trimer.

Immunogen engineering is a powerful tool to alter immunodominance hierarchies.^9^ These results suggest that caution is needed to avoid off-track B-cell engagement when changing immunogen designs within sequential immunizations. In particular, immunodominance hierarchies of epitopes presented on different designs may not be similar. This consideration may also be relevant to hybrid immunogens targeting multiple viruses (e.g., influenza and SARS-CoV-2) and polyvalent preparations aimed at inducing balanced responses against multiple virus serotypes (e.g., Dengue virus). Conversely, these results also suggest that, should B-cell repriming be desirable (e.g., to target immunodominant epitopes introduced in highly mutating viruses through natural evolution) pre-existing immunity against other epitopes should not constitute an insurmountable obstacle.

Finally, highly mutating viruses are known to escape humoral immunity by either shielding or directly mutating neutralizing epitopes (e.g., HIV-1, SARS-CoV-2, influenza antigenic drift), or by large envelope rearrangements (e.g., influenza antigenic shift). Our findings raise the intriguing, albeit speculative, hypothesis that the introduction of new immunodominant epitopes on their Env could suffice to redirect the immune response away from potentially protective epitopes, despite them being expressed on the virion surface, and act as an additional mechanism of immune evasion.

## Methods

### NHP multivalent immunization with a multiclade Env formulation.

The four NHPs included in this study (RNs14, RVv15, RPt15 and RSf15) were selected from an NHP trial designed to evaluate the effect of adjuvants in a DNA prime/polyvalent protein boost immunization strategy. Briefly, 40 NHPs were divided into 4 groups. One control group received mock immunizations. All other groups were primed three times (weeks 0, 4 and 8) with polyvalent HIV-1 gp120 envelope DNA (2 mg/construct) either without adjuvant (Group A), with pIL-12 (Group B) or with pMec/CCL28 (Group C). The polyvalent DNA preparation included the following DNAs: A.Q23.17, A.Q259d2.17, B.WITO4160.33, B.CAAN5342.A, B.THRO4156.18, C.Du172.17, C.ZM214M.PL15, and clade A, B and C consensuses. The first two immunizations were delivered i.d. with electroporation and the third immunization was delivered i.m. with electroporation. All groups were boosted twice i.m. (weeks 12 and 16) with 0.2 mg of a tetravalent gp120 Env protein cocktail (50 μg/protein) comprising clades A (92UG037.1), B (JR-FL), C (93MW965.26) and CRF AE (gp120 AE consensus) HIV-1 strains in Monophosphoryl Lipid A (synthetic) PHAD-(MPLA) adjuvant (0.4 mg). PBMCs were collected 14 days after the last immunization. All immunizations were delivered in the right thigh. NHPs were then challenged weekly up to 15 times with heterologous clade C SHIV.CH505.375H.dCT strain. All immunized NHPs but RVv15 (Group A) seroconverted. RNs14 and RPt15 (Group A) seroconverted at the second challenge, and RSf15 (Group B) seroconverted at the fourth challenge. Two NHPs in the mock group also resisted infection (not shown). The rhesus macaques used in this study were housed at Emory University and the study was approved by the Emory University Institutional Animal Use and Care Committee (PROTO201800112).

### Plasma viral load determination.

Quantitative real-time reverse-transcriptase (RT)-PCR assay to determine SHIV.CH505.375H.dCT viral load was performed as previously described.^10^ The sensitivity of the assay is 60 copies/ml of plasma.

### NHP sequential immunization with IDLV expressing CH505 Envs.

The study design has been previously reported in detail.^4^ Briefly, NHPs were immunized at 6-month intervals with non-stabilized gp140 Envs isolated from individual CH505, including the transmitted founder virus and sequentially evolved Envs isolated from weeks 53, 76 and 100 selected based on their binding profile to monoclonal antibodies of the CH103 bnAb lineage at progressive maturation stages (CH505.T/F, CH505.w53, CH505.w76, CH505.w100, respectively). Two additional immunizations were performed using stabilized CH505.w136 SOSIP Env trimer. Immunogens were delivered either through IDLV alone (3×10^8^ TU i.m.) or in combination with the respective protein (100 μg s.c.) in GLE-SE adjuvant. Six weeks after the last immunization, NHPs were challenged intrarectally with the autologous SHIV.CH505.375H.dCT strain. PBMCs were collected 14 days after the last immunization with CH505.w76 gp140 (week 75) and CH505.w100 SOSIP (week 117). The rhesus macaques used in this study were housed at BIOQUAL, Inc. and the study was approved by the BIOQUAL Institutional Animal Use and Care Committee (Study # 18–001).

### Memory B-cell phenotyping and sorting.

The following markers were used to define memory B-cells: CD20 FITC (BD Biosciences, catalog no. 347673, clone L27); IgD PE (Southern Biotech, catalog no. 2030–09); CD16 PE-Cy7 (BD Biosciences, catalog no. 557744, clone 3G8); CD27 APC-Cy7 (BioLegend, clone O323, catalog no. 302816); CD14 BV570 (BioLegend, catalog no. 301832, clone M5E2); and CD3 PerCP-Cy5.5 (BD Biosciences, catalog no. 552852, clone SP34–2). All antibodies were titered in advance and used at optimal concentrations. Memory B-cells were defined as follows: CD3^neg^/CD14^neg^/CD16^neg^/CD20^pos^/CD27^all^/IgD^neg^. To sort immunogen-specific memory B-cells, each gp120, gp140 or SOSIP trimer Env immunogen used in the respective NHP studies was tagged with either AlexaFluor 647 (AF647) or Brilliant Violet (BV412). For each study, all the pertinent Envs tagged with both fluorochromes were combined for staining. Memory B-cells positive for both the AF647- and BV412-tagged Env immunogens were sorted using a BD FACSAria II (BD Biosciences, San Jose, CA) and cultured as described below.

### Memory B-cell cultures.

We have previously described the memory B-cell culture method adapted to induce proliferation and differentiation of Rhesus memory B-cells into antibody-secreting cells.^3^ Briefly, memory B-cells were sorted as described above in bulk into wells containing 5000 MS40L feeder cells, RPMI-1640 supplemented with 15% FBS, 1 mM sodium pyruvate, 1% non-essential amino acids, 25 mM HEPES buffer, 2.5 μg mL^−1^ ODN2006 (Invivogen, Cat. no. TLRL-2006–5), 5 μM CHK2-inhibitor (Calbiochem, Cat. no. 220486–1MG), 100 ng mL^−1^ recombinant human interleukin (IL)-21 (Peprotech, Cat. no. 2001–21), 10 ng mL^−1^ recombinant Human BAFF (Peprotech, Cat. no. 310–13), 200 U mL^−1^ IL-2 (from the myeloma IL-2 producing cell line IL2-t6, kindly provided by Dr. Antonio Lanzavecchia, IRB, Bellinzona, Switzerland), and 100 μL supernatant of the Herpesvirus papio (HVP)-infected Baboon cell line S594 (NHP Reagent Resource). The concentration of each supplement was previously determined to achieve optimal in vitro stimulation. Following overnight incubation at 37°C in 5% CO_2_, memory B-cells were plated at limiting dilution in round bottom tissue culture 96-well plates containing 5000 non-irradiated MS40L feeder cells and cultured for 2 weeks. Culture medium was refreshed after 7 days and harvested 7 days later.

### ELISA culture supernatant screening.

High-binding 384-well plates were pre-coated with streptavidin for biotinylated antigens and coated with 2 μg mL^−1^ of Env proteins or 0.5 μg mL^−1^ of polyvalent goat anti-human Ig Ab (Life Technologies, Cat# H17000) to measure IgG, IgA, and IgM levels, diluted in 0.1 M NaHCO_3_ solution. Culture supernatants were tested at 1:3 dilution in blocking buffer. After two washes with washing solution, secondary HRP-conjugated antibodies (Jackson ImmunoResearch, Cat. no. 109-035-098, 109-035-129, and 109-035-011) were added at lot-specific optimal concentrations for 1 h. After 4 washes, plates were developed for 10 min using 15 μL per well SureBlue Reserve TMB microwell peroxidase substrate before adding 15 μL per well of 0.1 M HCl. All of the following criteria had to be met to define culture supernatant positivity for binding to Env immunogens: measurable IgG, IgA, or IgM levels; OD_450_ > 0.1 and > 2 × OD_450_ reads from blank wells; OD_450_ > 120% OD_650_.

### HIV-1 gp120 Env phylogenetic analysis.

The unrooted phylogeny of the four HIV-1 gp120 protein sequences was calculated using the maximum likelihood algorithm implemented in IQtree-2. The same topology with similar branch lengths was also calculated using the Bayesian phylogenetic analysis program MrBayes.

## Figures and Tables

**Figure 1.**
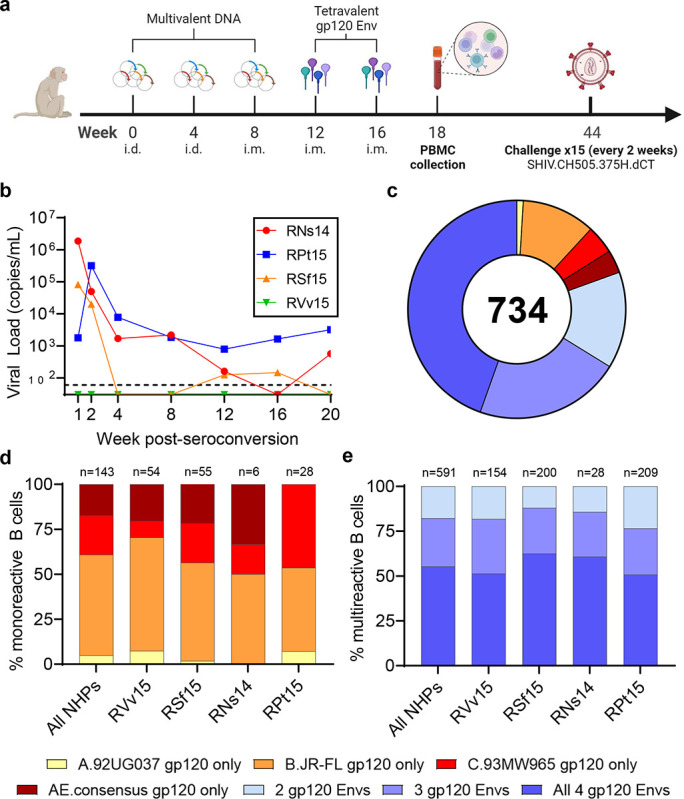
Tetravalent gp120 Env immunization. **a** NHP immunization scheme with multivalent HIV-1 gp120 Env cocktails delivered as DNA and monomeric proteins. **b** Viremia time-course of the four NHPs included in this study post-seroconversion or after the fifteenth challenge for RVv15. **c** Immunogen binding profiles of 734 B-cells isolated from all four NHPs. Reactivity with individual and multiple gp120 Env immunogens is color coded as indicated. **d** Binding to individual immunogens of monoreactive B-cells, expressed as percentage of the total number of monoreactive B-cells. Data are shown as aggregate (“All NHPs”) and for each NHP. **e** Immunogen cross-reactivity of multireactive B-cells, expressed as percentage of the total number of multireactive B-cells, shown as aggregate (“All NHPs”) and for each NHP.

**Figure 2.**
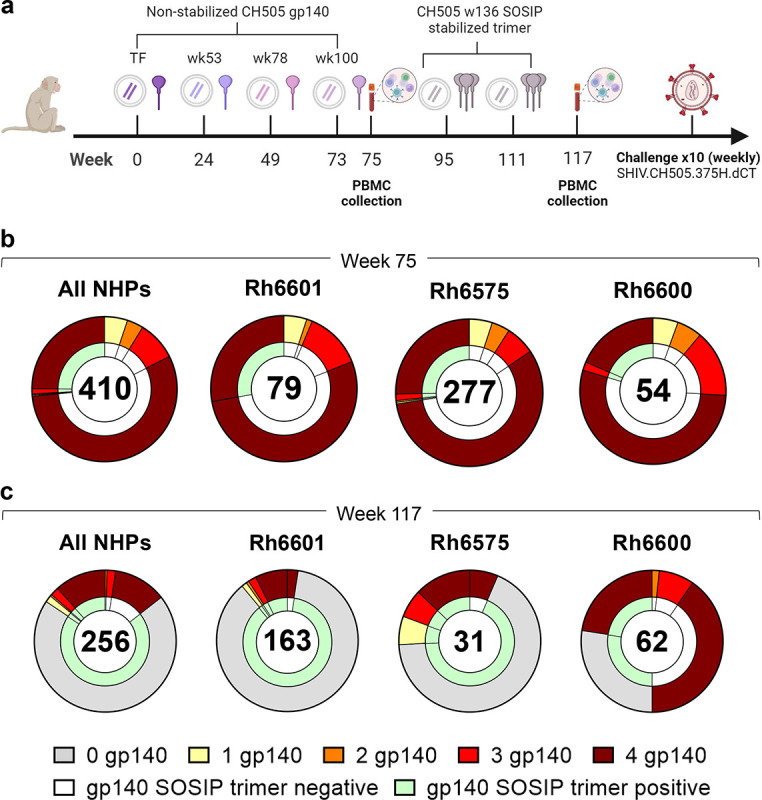
Sequential immunization with clonally related Envs. **a** NHP immunization scheme (see methods and ref^4^). **b,c** Cross-reactivity of B-cells isolated **(b)** before and **(c)** after SOSIP trimer immunizations with non-stabilized gp140 Envs immunogens (outer ring) and stabilized CH505.w136 SOSIP trimer (inner ring). Data are shown as aggregate (“All NHPs”) and for each NHP. Number of B-cells analyzed is shown for each chart. Reactivity with one or more non-stabilized gp140 Env immunogens is color coded as indicated. Absence of binding to any of the four non-stabilized gp140 Env immunogens is shown in gray.

## Data Availability

The datasets generated and analyzed in this study are available from the corresponding author on reasonable request.
